# Radiopharmaceutical small-scale preparation in Europe: will we be able to harmonize the situation?

**DOI:** 10.1186/s41181-024-00281-z

**Published:** 2024-09-05

**Authors:** Estrella Moya, Celia Cerrato, Luis Miguel Bedoya, José Antonio Guerra

**Affiliations:** 1 Chemical and Pharmaceutical Technology Division, Medicines for Human Use Department, AEMPS: Agencia Española de Medicamentos y Productos Sanitarios, C/ Campezo nº 1, Edificio 8, Madrid, 28022 Spain; 2 Department of Pharmacology, Pharmacognosy and Botany, Faculty of Pharmacy, Universidad Complutense de Madrid, Plza. Ramón y Cajal s/n, Madrid, 28040 Spain; 3 AIDS Immunopathology Department, National Centre of Microbiology, Instituto de Salud Carlos III, Ctra. Pozuelo Km. 2, Madrid, 28224 Spain

**Keywords:** Radiopharmaceuticals, Regulation, Quality requirements, Small-scale production, EMA guidelines, Regulatory framework, European union

## Abstract

**Background:**

Radiopharmaceuticals have been considered a special group of medicines in Europe since 1989. The use of radiopharmaceuticals that have marketing authorization should always be the first option in clinical use, however due to their special properties the availability of approved radiopharmaceuticals is limited. For this reason, they can be produced on a small scale outside the marketing authorization process.

**Main body:**

The in-house radiopharmaceutical preparations represent an important source of these special medicines for routine nuclear medicine practice. However, a lack of harmonization in Member States’ regulations leads to extreme differences in the use and availability of radiopharmaceuticals across Europe. The aim of this work is to provide an overview of the different national regulatory frameworks in which Directive 2001/83/UE is adopted on the preparation of radiopharmaceuticals outside the marketing authorization track in Europe. Nine different national regulations have been studied to describe how unlicensed radiopharmaceuticals are prepared. Special attention is paid to reflect the minimum standards that these preparations should meet as well as the educational requirements to be a radiopharmacist in charge of them.

**Conclusion:**

The rapid development of new radiopharmaceuticals used in radiometabolic therapy requires a common regulation that allows balance between the use and preparation of licensed and unlicensed radiopharmaceuticals. The absence of a harmonized regulation for the radiopharmaceutical small-scale preparation and the implementation of Good Manufacture Practices, leads to extreme differences in the use, quality assurance and availability of radiopharmaceuticals in Europe.

**Supplementary Information:**

The online version contains supplementary material available at 10.1186/s41181-024-00281-z.

## Background

The field of Nuclear Medicine relies heavily on radiopharmaceuticals (RPs), which are substances containing radioactive isotopes used for diagnostic and therapeutic purposes. RPs have been considered medicinal products in the European Union since 1989, and are currently regulated by Directive 2001/83/ EC, the Community code relating to medicinal products for humans [1].

These radiopharmaceuticals are either acquired from commercial suppliers or prepared in-house for immediate use. In-house preparations, although non-commercial, play a significant role in routine Nuclear Medicine practices for both diagnostic and therapeutic applications. The use of RPs that have marketing authorization should always be the first option in clinical use. However, due to their special properties (very short shelf life), the availability of approved radiopharmaceuticals is limited. For this reason, the in-house radiopharmaceutical preparations represent an important source of these special medicines for routine nuclear medicine practice [2, 3].

European legislation, specifically directive 2001/83/EC, mandates that commercially distributed RPs must obtain marketing authorization (MA) to be legally marketed [1]. However, the availability of such authorized RPs is limited due to their special nature and the limited market potential for those used in rare clinical cases [4].

Innovation in RPs often originates in radiopharmacies, research institutes, or nuclear medicine labs. Many ground-breaking developments in Nuclear Medicine in recent years, such as the success of theragnostic with somatostatin analogues and prostate cancer treatments, were based on in-house preparations of innovative RPs [5–7]. When a new RP shows both technical and clinical potential for commercial distribution, pharmaceutical companies usually take over from academia to fund further clinical trials [8].

European legislation treats RPs differently from non-radioactive pharmaceuticals. A marketing authorization is required not only for ready-to-use RPs but also for starting materials like radionuclide generators, radionuclide precursors and kits. These starting materials, with a marketing authorization, are referred to as “licensed” [9, 10].

The rate and extent of adoption and interpretation of the Directive varies from country to country, and as a result, several European Member States have established a legal framework in which RPs can be prepared on-site for routine use, without the need for marketing authorization. These exemptions derive from the definitions in Article 3 of Directive 2001/83/ EC, the so-called magistraland officinal formulas, and from Article 5(1) of Directive 2001/83/ EC, which aims to meet specific needs [1].

Besides, the European Pharmacopoeia (Ph. Eur 2619) [11] describes ethical considerations and guidelines for the manufacture of unlicensed pharmaceutical preparations. In this case, all healthcare professionals involved have a duty of care individualized for the patient receiving the medicine, when deciding whether to use an unlicensed preparation within their scope of responsibility. In addition, an appropriate risk assessment should be performed. In this way, the criticality of various parameters (e.g., quality of active ingredients, excipients, etc.) and the risk that the preparation may pose to a particular patient population, should be considered.

The aim of this paper is to analyse nine national regulations (France, Italy, Belgium Germany, Denmark, Austria, Finland, United Kingdom and Spain) in which Directive 2001/83/EC has been transposed. Special attention needs to be paid to the minimum standards that these preparations should meet, the regulatory body in charge of granting these special permits as well as the educational requirements to be a radiopharmacist in charge of them.

This review discusses the current legislation in these European Union countries regarding the non-commercial, in-house preparation of RPs in compliance with both European and national regulations. It covers “compounding” which involves using licensed starting materials, as well as the preparation of diagnostic (Positron Emission Tomography (PET) and Single Photon Emission Tomography (SPECT)) and therapeutic RPs using more complex methods and usually unlicensed starting materials. The review references guidelines outlined in the current Good Radiopharmaceutical Practice (cGRPP) as a basis for recommendations in this field.

The current routine practice in nuclear medicine is still mainly driven by “in-house” radiolabelling kits, where radionuclides are manually added by the hospital radiopharmaceutical units in sterile conditions under radio safety precautions to prepare the final radiopharmaceutical compounds closest to the patient use. This manuscript aims to provide a comprehensive review of the legislation in force in different EU countries on the requirements for facilities and personnel for the authorization of small-scale manufacture of radiopharmaceuticals, as well as for their subsequent marketing authorization.

## Main text

### European legislation frame on radiopharmaceuticals

The European harmonization process in the pharmaceutical field started in 1965 with the adoption of Directive 65/65/EEC [12]. This directive stated provisions for enhancing the access to the market to new drugs, including RPs. RPs were exempted from various aspects of the pharmaceutical legislation for a reasonably long period in Europe and kept with other exclusive products, including vaccines, blood-derived products and allergens [13].

Initially, rules primarily focused on radiation protection and pharmacopoeia compliance. However, Directive 89/343/EC marked a significant shift, extending existing medicinal product regulations to include RPs used for diagnosis and treatment [14]. EU member states have adopted this directive which requires Marketing Authorization (MA) for RPs that have been on the market for over 20 years. Manufacturers faced challenges as they were required to submit comprehensive dossiers with preclinical and clinical trial data to obtain MA. This change aimed to ensure the quality, safety and efficacy of radiopharmaceutical products in the market.

The preparation and use of RPs in the European Union are regulated by directives, regulations and rules that have been adopted by member states (Table 1). The rate and extent of adoption and interpretation of directives varies among countries and each member state may introduce changes, as long as the objective of the directive is met.

**Table 1:** *Summary of legally binding and guidance documents for radiopharmaceuticals in Europe* [3, 9, 15]

	Categories of radiopharmaceuticals		
	Marketing authorization	Clinical trials	In-house preparations
Legally binding documents	Ph. Eur. General and Specific Monographs		
	Directive 2001/83/EC	Directive 2001/20/EC	National governance
	Directive 2003/94/EC	Directive 2003/94/EC	
	Directive 2004/27/EC	Directive 2005/28/EC	
	GMP Annex 3	Regulation 536/2014	
		GMP Annex 13	
Guidance documents	EMA Guideline on Radiopharmaceuticals	EC Guidance IMP/NIMP	Ph. Eur. General Chap. 5.19
		EMA Guideline IMPD	PIC/S GPP 010 − 4 incl. Annex 3
		EMA Guideline first-in-human clinical trials	EANM guidelines and guidance documents
		EANM guidelines and guidance documents	National documents

EC: European Commission; EMA: European Medicine Agency; EANM: European Association of Nuclear Medicine; GMP: Good Manufacturing Practices; GPP: Guide to Good Practices; IMP: Investigational Medicinal Product; IMPD: Investigational Medicinal Product Dossier; PIC/S: Pharmaceutical Inspection Co-operation Scheme

The **small-scale** preparation of medicines is important in order to accommodate individual patients’ needs in Europe. This is, in particular, the case if an appropriate authorized medicine does not exist or is unavailable on the market. European Union (EU) regulation determines under which conditions a marketing authorization is required in order to place a medicinal product on the market and under which conditions a manufacturing and wholesale license is required.

The Directive 2001/83/CE regulates multiple aspects relating to drugs and RPs: MA, manufacture and import, distribution, advertising and pharmacovigilance. It contains provisions (e.g. articles 6 and 7) for MA for industrially manufactured RPs and their exemptions in certain cases for extemporaneous preparations, as small-scale production of RP [3, 4, 14].

RPs can be grouped into two categories: industrially manufactured and extemporaneously prepared RPs (Table 2). The latter ones are generally accepted only when an authorized industrial radiopharmaceutical is not available on the market.

**Table 2:** Regulatory sources for radiopharmaceuticals preparations

Industrially manufactured radiopharmaceuticals	Extemporaneously prepared radiopharmaceuticals
With marketing authorization • Directive 2001/83/CE	Officinal formula: • Directive 2001/83/CE, art 3 • National legislation
Without marketing authorization • Directive 2001/83/CE, art 5	Magistral formula: • Directive 2001/83/CE, art 3 • National legislation
Clinical trials • Regulation (EU) 536/2014 • Directive (UE) 2017/1572 (GMP regulation)	Clinical trials: • Regulation (EU) 536/2014

CE: European Commission; EU: European Union; GMP: Good Manufacturing Practices

### National regulation of radiopharmaceuticals

Many radiopharmaceutical preparations are prepared in small scale on-site regularly, often as a single dose for one patient, based on specific clinical needs usually called extemporaneously prepared radiopharmaceutical preparations (EPRPs). Commonly, EPRPs included: magistral formula (prepared in a pharmacy following a medical prescription) and officinal formula (that follows pharmacopoeia monograph) and both are intended to be supplied directly to the patients [3, 16]. In Europe, the regulations about EPRPs vary among the European countries as no directive is approved. Here nine countries were analyzed: France, Italy, Belgium, Germany, Denmark, Finland, Spain, Austria and the UK.

## France

The production of RP in France - as for all other medicinal products – strictly requires supervision by a pharmacist who is taking over the responsibility (“*monopole de pharmacien*”). France transposed Directive 89/343/EEC into the national legal framework by Law 92–1279/1992, which amended the French Public Health Code (Code de la Santé Publique). Le Code de la Santé Publique is considered the most important document within the French regulatory framework, in which the definitions of RPs in Art. L5121-1 of Directive 2001/83/ EC have been implemented [17] The Code distinguishes between public pharmacies (“*officines de pharmacie*”) and pharmacies for internal use (“*pharmacie à l’usage intérieur* ´PUI). PUIs are usually involved in the preparation of magistral preparations for a single patient. Unlicensed RPs may be considered as such and their preparation is allowed only in a PUI.

The Regional Health Agency (ARS, Agence Régionale de Santé) is the body responsible for granting site authorization for PUI. (Article L5126-4 of the Public Health Code) [17]. The authorization is specific to the type of preparations, in this case, an application for the preparation of radiopharmaceuticals is required and the type of activity to be performed diagnostic, curative and/or research should also be specified.

The person in charge of preparing the RP should be a radiopharmacist. In France, radiopharmacy is a specialty of pharmacy internship leading to a Diploma of Complementary Specialized Studies (DESC) of Radiopharmacy and Radiobiology, which comes in addition to the diploma of pharmacist former intern of hospital.

Radiopharmaceutical products must be prepared according to good practices of preparation (Bonnes Practiques de Préparation BPP) [18], in line with Article L. 5121-5 of the Public Health Code (CSP). The principles of this practice are laid down in a decision of the National Agency for the Safety of Medicines and Health Products (ANSM).

## Italy

Italy has transposed Directive 2001/83/ EC into the national legal framework through Legislative Decree No. 219 of April 24, 2006 [19]. The Agenzia Italiana del Farmaco (AIFA.) is responsible for pharmaceutical aspects, applications for marketing authorization and clinical trials, inspections of production sites and evaluation of dossiers. As in the other European countries, other institutions are responsible for the radiation protection aspects.

Non-authorized RPs may be prepared as a magistral formula and officinal formula under the exception specified in Article 3 of the above Directive. The quality requirements for magistral formulations are defined in the “Norme di buona preparazione in medicina nucleare” (Good compounding practice in nuclear medicine, NBP-MN) published in the Official Pharmacopeia of the Italian Republic [20]. Although NBP-MN is based on the same general principles of GMP, the special nature of RPs is considered.

The NBP-MN” states, “The preparation and quality control of RPs must be performed by qualified personnel who have sufficient knowledge to work with unsealed radioactive materials under controlled conditions.” However, apart from this general statement about adequate training and scientific expertise, there are no specific requirements for the education and training of personnel to become radiopharmacists. In this way, pharmacists, chemists, biologists and even nuclear physicians could be designated as qualified persons responsible for the release of the radiopharmaceutical, as stated in Article 52, paragraph 3b, of Leg. Decree 219/2006 [21]. In addition, two years of practical experience in approved establishments for the manufacture of medicinal products should be demonstrated.

## Belgium

Belgium transposed Directive 2001/83/EC into its national legal framework through the Law of March 25 1964 on medicinal products for human use (Loi sur les médicaments à usage humain) [22]. Radiopharmaceutical products prepared outside the marketing track can be considered as magistral formulas. Accordingly, they are prepared in authorized hospital pharmacies in accordance with the Royal Decree of September 30, 2020 [23].

The requirements for the manufacturing of pharmacy preparations are set forth in Article 22 of the mentioned Royal Decree. The registration of a hospital pharmacy is mentioned among these requirements. In addition, the presence of a hospital pharmacist, in our case the radiopharmacist who will be responsible for the preparation of the RPs, is mandatory. The requirements for a qualified person are specified in Article 84 of the Royal Decree of December 14, 2006, and no specific requirements are described in the case of RPs [24].

The quality requirements to be met by magistral formulas are described in the guide to good pharmacy practices (Guide des bonnes pratiques pharmaceutiques officinale) published on the website of the Agence Fédérale de Médicaments et des Produits de Santé (AFMPS) and in the Guide to good practices for the preparation of Medicinal Products in Healthcare Establishments published by The Pharmaceutical Inspection Co-operation Scheme (PIC/S) [25] in accordance with the provisions of the Royal Decree of September 2020 [23]. For RPs, a specific section exists for (PIC/S) radiopharmaceutical manufacturing.

### Germany

The Medicinal Products Act (Arzneimittelgesetz – AMG), published on May 16, 1961[26], is the main legal framework for the regulation of medicinal products in Germany. It has been amended to implement the Directives of the European community. In the AMG Sect. 7 (§ 7) the dispensing of radioactive medicinal products is prohibited, unless there is a permission by legal regulation according to para. 2 (§ 7.2). The Directive 2001/83/EC was translated in the § 7 AMG with the ‘Ordinance on Radioactive Medicinal Products and Medicinal Products Treated with Ionizing Radiation’ (Verordnung über Radioaktive oder mit ionisierenden Strahlen behandelte Arzneimittel, AMRadV) [27]. However, some differences can be found in the German regulatory framework in comparison to the other European Countries. For example, it does not include a specific definition for a radiopharmaceutical kit. AMRadV allows the preparation and distribution of unlicensed RPs for diagnostic purposes that are prepared by a clinical facility. Legal/local authorities will grant the manufacturing authorization to these sites, which will be subject to regular GMP inspections.

The number of applications of a diagnostic radiopharmaceutical is limited to a number of 20 patients per radiopharmaceutical per week on-site. For more than 20 doses of a radiopharmaceutical per week, a marketing authorization is required, which then also allows distribution of the radiopharmaceutical to other sites and is subject to regular GMP inspections.

For unlicensed therapeutic RPs, the preparation of which is not covered by the above regulations, the AMG (§ 13. 2b) permits radiopharmaceutical preparation, provided that it is carried out under the full responsibility of a physician and is prepared for a single patient (§ 13. 2b) [27].

The preparation should be made according to state-of-the-art pharmaceutical standards, i.e. the European Pharmacopoeia, if monographs are available. It should be noted that this regulation is also used in many university hospitals for other investigational unlicensed RPs.

However, it is important to note that Germany is a federal republic, in which local state authorities may vary drug production and drug compliance controls from state to state.

Regarding the training of personnel, there is no formal recognition as a radiopharmaceutical specialist in Germany, and the requirements for a qualified person in facilities that manufacture RPs are listed in AMG § 15 [27]. In summary, the qualified person in charge of the manufacture of RPs must have a degree in natural sciences or medicine and 3 years of practical experience in nuclear medicine or radiopharmaceutical chemistry. These requirements differ from those for the manufacture of other medicinal products, where only two years of practical experience in the quality control of medicinal products is required in addition to a license to practice as a pharmacist or postgraduate training equivalent to pharmacy studies.

### Denmark

The Directive 2001/83 EC has been transposed into the Danish Medicines Act, which contains provisions on the authorization and control of medicinal products [28], including radiopharmaceutical, by the Danish Medicines Agency. On its webpage is published an application for handling RPs in hospitals, where it is also depicted that if hospitals want to prepare RPs from raw materials, a GMP manufacturing authorization is required. This application is in accordance with the provisions of the Danish Medicines Act § 39 [29] and the Executive Order No 1231 of 12 December 2005 on radiopharmaceuticals [30].

Generally, hospitals and PET centers are not allowed to produce RPs from raw materials when there is already a radiopharmaceutical preparation with a marketing authorization available on the market. However, in cases of non-availability of RPs preparations, hospitals/PET-centers can produce them as long as they are granted with a special permission for compassionate use. This permission requires the submission by the interested party (hospital/PET-center) of a set of quality documentation for approval by the Danish Medicines Agency. These centers have to comply with GMP and regular inspections of these sites are programmed.

There are no specific formal requirements for qualification as a radiopharmacist/radiochemist in Denmark. However, a course that provides basic training in radiochemistry is offered at Copenhagen University [31]. The requirements to be a Qualified Person are the same as those needed for conventional pharmaceuticals.

### Austria

Austrian Medicines Act - AMG 1983 [32] regulates the medicinal products manufacture and placing on the market. This Regulation therefore covers all aspects related to the authorization of RPs.

As in the rest of European countries, radiation protection and pharmaceutical aspects in the preparation of RPs are encompassed within the Member State regulatory responsibilities. In this case, the Austrian Agency for Health and Food Safety (AGES) jointly with Federal Ministry of Health and the associated Austrian Federal Office for Safety in Health Care (BASG), are the authorities in charge of the pharmaceuticals aspects [33].

The particularities for the use of unlicensed medicinal products, especially with unlicensed RPs, shall be taken into consideration according to the Federal Office for Safety in Health Care (BASG) [34]. Consequently, although radiopharmaceutical products need to be granted with a marketing authorization as a general rule in Austria, unlicensed RPs products could still be dispensed within the framework of the two following exemptions stated in the Austrian Medicines Act [32]:

- First exception is based on disposition § 7 Abs. 8 of the AMG; so-called “notice of reduced quantities”. The situation, described in this provision, is not an alternative to available authorized RPs. Moreover, they should be used in small quantities and provided that all the requirements according the above-mentioned disposition are met.
- Second exception is detailed on disposition § 8 Abs 1 Z 2 AMG; they can be prepared as a radiopharmaceutical magistral formula under the prescription of a nuclear physician provided that, there is a direct and immediate use (in-house only) in hospital radiopharmacy.

Regarding the mandatory authorization for radiopharmacy units, they could be exempted according to article § 62 in AMG (*Manufacturing Sites of Pharmaceuticals Operating Regulations*), since laboratories that manufacture RPs exclusively for the purpose of direct use to patients are not required to be authorized radiopharmacy units [32].

The quality requirements for authorized medicines according to European directives EUDRALEX are implemented in the Austrian GMP Directive [35]. However, for the “in-house” preparation of RPs, Guidance on current good radiopharmacy practice (“cGRPP”) of the EANM [2], as well as the PIC/S Guideline for Healthcare Establishments [25] along with the European Pharmacopoeia, can be considered as appropriate [36].

Concerning staff training, there is no formal recognition in Austria as radiopharmacist specialist and the requirements to be a Qualified Person are the same applicable to conventional pharmaceuticals.

### Finland

In Finland the legislation relating to pharmaceuticals is largely based on EU regulations. Therefore, the Medicines Act (395/1987) and the Medicines Decree (693/1987) regulate the manufacturing, import, authorization, distribution, and selling of medicinal products [37, 38]. The regulatory authority in charge of licensing, supervision, and regulatory activities related to human medicines and the pharmaceutical sector is the Finnish Medicines Agency (Fimea).

The use of medicinal products granted with a marketing authorization is always the primary option in patients’ pharmacotherapy. However, in individual cases and for special therapeutic reasons, Fimea may authorize the release for consumption of a medicinal product that has no marketing authorization in Finland. A special permit is granted for up to one year based on a situation and case-specific overall assessment (Medicines Act Sect. 21) [37]. This is also applicable to RPs. The special permit procedure is limited to use in exceptional cases where no other treatment is appropriate or yields the desired effect [38].

Radiopharmaceutical industrial manufacturing requires a Manufacturing Importation Authorization (EU MIA; Finnish Medicines Act 8§). However, hospital pharmacies may also manufacture RPs (Medicines Act 14, 61, 62§), but only for limited use i.e. to the extent required by hospital district, hospital or health center operations or operations referred to in Sect. 62/3 [38].

Regarding compliance with Good Manufacturing Practices (GMP), hospital pharmacies and medicines dispensaries are required to observe the principles of best manufacturing practices when producing medicines. The PIC/S guide will offer support and guidance to professionals in implementing this requirement in everyday practice [25].

Concerning staff training, there is no formal recognition in Finland as radiopharmacist specialist, and the requirements to be a Qualified Person in institutions, which produce RPs, follow the general EU rules for QP for human medicines [1].

### Spain

Spain transposed Directive 2001/83/EC into the national regulatory framework by Royal Legislative Decree RDL 1/2015 where RPs are included in the article 48 [39]. The preparation of RPs cannot be considered as a magistral formula since they must be prepared with legally recognized action and indication substances as stated in the mentioned RDL. Therefore, preparing PET RPs outside the marketing authorization track would be under the regulatory framework of article 47.1c in Real Decree (RD) 1345/2007 on the marketing authorization procedure in medicines for human use [40]. This Decree completes the RDL 1/2015. Article 47.1.c in the RD 1345/2007 establishes the following criteria: “*The marketing authorization for PET RPs will not be required, whenever they are prepared in an approved radiopharmacy unit under the supervision and control of a radiopharmaceutical specialist, provided that they meet the following requirements*” [41]:

1. They are entirely prepared and used in authorized radiopharmacy units with non-profit use and in centers linked to the National Health System.
2. They are substances used in clinical research or medicines that the Spanish Agency for Medicines and Medical Devices (AEMPS) considers to satisfy the guarantees of quality, safety, efficacy, identification, and information, and that are prepared in appropriate facilities.

The Spanish Agency of Medicines and Medical Devices (AEMPS) will grant a certificate for each radiopharmaceutical individualized preparation, therefore, radiopharmacy units should submit an application with all the information required. Additionally, the radiopharmacy unit should be previously inspected by AEMPS Inspection Department. The information required (clinical, non-clinical, and quality), will depend on the radiopharmaceutical situation with or without EMA-Core summary of product characteristics or if the radiopharmaceutical product is already authorized in the European Union or not.

In this way, to prepare a non-authorized radiopharmaceutical without core-SPC all the information related to clinical, non-clinical, and quality should be submitted.

As it stated in article 48 of RDL 1/2015 the person in charge of the preparation should be a radiopharmacist specialist. The educational requirements to obtain this specialization are 3 years of practical experience in a university hospital [39]. The requirements to be a QP stated in Art 16 of RD 824/2010 are the same as the conventional pharmaceuticals [42].

### United Kingdom

Medicines & Healthcare Products Regulatory Agency (MHRA) in accordance with the Human Medicines Regulations 2012 (SI 2012/1916) undertakes the regulation of medicines on the UK market [43]. . Unlicensed RPs can be prepared as “specials”, applying the exception to Art. 5(1) of the Directive 2001/83/EC, regulated when The United Kingdom was still a member state of the European Union [1].

Regulation 167 of the Human Medicines Regulations 2012 sets out the exemption from the requirement for a medicinal product, placed on the market in the UK, to hold a marketing authorization. Additionally, the MHRA issued Guidance Note 14 called “The supply of unlicensed medicinal products “(“specials”) [44]. The manufacturer of “specials” must hold a Manufacturer’s “Specials” License granted by the Licensing Authority and the manufacturing site and its operations will be inspected for compliance with Good Manufacturing Practice (GMP) and the conditions of the license.

Pharmacists in hospitals are authorized to procure specials in the UK. It is necessary to have a head of production (Production Manager) and a different independent person responsible for quality control (Quality controller) [45].

There is a formal route to be a radiopharmacist in the UK through a Master of Science run by one university. This M.Sc. allows the registration as a clinical pharmaceutical scientist and then undertaking an extended placement in the radiopharmacy area [46].

### Current comparative status of the legislative framework

Within the European Union and the United Kingdom, there are four different ways to carry out the RPs small-scale preparation outside the marketing authorization track. Depending on the national legislation in each Member State, the consideration will vary (Table 3):

1. In some Member States (France, Italy, Belgium) the unlicensed small-scale radiopharmaceutical preparations follow Article 3 of Directive 2001/83/EC and they can be considered as magistral formulas and officinal formulas.
2. In the United Kingdom the exception of art 5(1) of Directive 2001/83/EC is applied and unlicensed small-scale radiopharmaceutical preparations are considered as special products.
3. In Spain, Denmark, and Finland, unlicensed RPs can be prepared provided that special permits are granted according to their respective national regulations.
4. By contrast, in Austria and Germany each situation can be treated differently. In some cases, unlicensed RPs could be considered as magistral formulas and in other cases, they follow their national legal framework that allows their preparation.

Analyzing compliance with Good Manufacturing Practices (GMP), various degrees of compliance have been observed. For example, in some Member States such as France and Italy adapted GMP, known as Good Compounding Practices, are enforced. In other Member States, such as Belgium, Austria, and Finland, the requirements for the manufacture of RPs are met according to Annex 3 of the Pharmaceutical Inspection Co-operation Scheme (PIC/S). Finally, in the other Member States (Spain, Germany, Denmark) and the United Kingdom, full GMP are mandatory.

**Table 3:** Summary of the national regulation on radiopharmaceuticals small scale preparation

Country	RPs as magistral formula/officinal formula	RPs to fulfil special needs	RPs regulated by national laws	Compliance with GMP	Compliance with other guidelines	Regulatory agency	Regular Inspections
AT	Yes	NO	Yes	NO	Guidelines (“cGRPP”) of the EANM PIC/S	BASG	Yes
BE	Yes	NO	Yes	NO	PIC/S	AFMPS	NC
DK	NO	NO	Yes	Yes	Not	DMA	Yes
FI	NO	NO	Yes	NO	PIC/S	FIMEA	NC
FR	Yes	NO	Yes	NO	BPP	ASNM	NC
DE	NO	NO	Yes	Yes	Yes	BfArM	Yes
IT	Yes	NO	Yes	NO	NBP-MN	AIFA	NC
ES	NO	NO	Yes	Yes	NO	AEMPS	Yes
UK	NO	Yes	Yes	Yes	NO	MHRA	Yes

AT: Austria; BE: Belgium; DK: Denmark; FI: Finland; FR: France; DE: Germany; IT: Italy; ES: Spain; UK: United Kingdom. BASG: Bundesamt fYesr Sicherheit im Gesundheitswesen (Germany Medicines Agency); AFMPS: Agence fédérale des médicaments et des produits de santé (Belgium Medicines Agency); DMA: Danish Medicines Agency; FIMEA: Finnish Medicines Agency; ASNM: Agence nationale de sécurité du médicament et des produits de santé (French Medicines Agency); BfArM: Bundesinstitut fYesr Arzneimittel und Medizinprodukte ( Federal Institute for Drugs and Medical Devices); AEMPS: Agencia Española del Medicamento y Productos Sanitarios (Spanish Medicines Agency); AIFA: Agenzia Italiana del Farmaco (Italian Medicines Agency); MHRA: Medicines and Healthcare products Regulatory Agency. GMP: Good Manufacturing Practices; cGRPP: Guidelines on current good radiopharmacy practice; EANM: European Association of Nuclear Medicine; BPP: Bones Practiques de Préparation; PIC/S: Pharmaceutical Inspection Co-operation Scheme; NBP-NM: Norme di Buona Preparazione in medicina nucleare. NC: Not confirmed

Concerning the training required to be a radiopharmacist, it is noted that in most of countries, there is no formal certificate to be considered a radiopharmacist specialist. Therefore, different pathways to reach the needed training have been observed. For instance, in the United Kingdom, Denmark, and Germany, curricula for the completion of basic training in radiopharmacy are offered in some universities In addition, there is a complete postgraduate training programme in radiopharmacy, consisting of three modules, organized by the ETH Zurich (together with the University of Ljubljana and the University of Leipzig). Upon completion of the three modules (the first in Pharmacy and Legislation, the second in Radiopharmaceutical Chemistry, and the third in Radiopharmacology and Clinical Radiopharmacy), graduates are awarded the ETH Certificate of Advanced Studies (CAS) in Radiopharmacy. This course is taken by many people in Germany to obtain a certificate in radiopharmacy and is mandatory to work as a QP for RPs in Switzerland.

The ETH courses are recognized by the EANM, which may issue the postgraduate certificate to candidates who have successfully completed them and obtained the corresponding CAS, but who must also demonstrate that they have completed a two-year period of experience in a radiopharmacy department, during which they have completed the practical components of the ETH course syllabus, and have completed a nationally recognized course in radiation safety”.

On the other hand, in the case of Spain and France, there is an official certificate to be considered a radiopharmacist specialist. In both countries, a specializationin radiopharmcy is obtained through a period of practical experience in radiopharmacy in hospitals and in the radiopharmaceutical industry (3 years in Spain and 2 years in France). Other certificates mentioned above are not legally recognized in these countries.

The lack of harmonization, concerning RPs small-scale preparations and GMP implementation, leads to extreme differences in the use, quality assurance, and availability of RPs in Europe. Thus, in some countries, a wide variety of RPs can be prepared in the hospital, including RPs for diagnosis and therapy, while in other countries preparations are possible only for diagnosis RPs and in limited cases.

Since small-scale preparation is subject to national laws, there seems to be no easy solution, but authorities should try and find a way to manage this challenging situation in order to harmonize, across European countries, the radiopharmaceutical small-scale preparations for the benefit of patients.

However, the use of approved RPs should not be completely disregarded considering the large investments of the radiopharmaceutical industry to obtain marketing authorization for some extensively used RPs. This ensures the availability of an adequate supply of these essential medicines, produced under high-quality standards.

In this context, and in line with the findings of Patt et al. (2023), it is evident that despite multiple revisions, the community legislation pertaining to radiopharmaceuticals has remained largely unchanged since its inception in 2001[47]. Nevertheless, this landscape has since evolved significantly. Notably, there has been a growing emphasis on in-house production, now permitted through specific national exemptions from Directive 2001/83/EC. This shift has resulted in substantial diversity in nuclear medicine practices and the availability of radiopharmaceuticals throughout Europe. Despite the remarkable progress in the field of nuclear medicine, which has had a clearly positive impact on patient care, particularly with the successful emergence of theranostics for both cancer diagnosis and treatment, radiopharmaceuticals have not been a central focus of the European Commission’s pharmaceutical strategy.

However, since the preparation and administration of these medicines ultimately depends on the radiopharmacist and the medical personnel, their proper training must not be overlooked under any circumstances. Although radiopharmacist training in Europe does not seem to be standardized, it has been found that, as a general rule, radiopharmacists in Europe acquire adequate skills and sound knowledge for the preparation of RPs with high quality standards.

## Conclusions

Overall, the lack of harmonization regarding small-scale radiopharmaceutical preparations and the implementation of Good Manufacturing Practices (GMP) leads to significant disparities in the utilization, quality assurance, and availability of radiopharmaceuticals across Europe. Although there is no straightforward solution due to small-scale preparations being subject to national legislation, authorities should strive to find a means to address this challenging situation in order to standardize radiopharmaceutical small-scale preparations across European countries for the benefit of patients. It is suggested that achieving a balance between the use of licensed and unlicensed radiopharmaceuticals would be a mutually beneficial solution for nuclear medicine services, the industry, and ultimately, patients. Furthermore, emphasis is placed on the importance of ensuring proper training for the medical and pharmaceutical personnel involved in the preparation and administration of these medications. Failure to implement such legislation may result in continued discrepancies in patient access to quality radiopharmaceuticals, potentially compromising the effectiveness of nuclear medicine services and patient care outcomes across Europe.

## Electronic supplementary material

Below is the link to the electronic supplementary material.

Supplementary Material 1

## Data Availability

Data availability is not applicable to this article as no new data were created or analysed in this study.

## Abbreviations

AEMPS: Spanish Agency of Medicines and Medical Devices
AFMPS: Agence Fédérale de Médicaments et des Produits de Santé
AGES: Austrian Agency for Health and Food Safety
AIFA: Agenzia Italiana del Farmaco
AMG: Medicinal Products Act (Arzneimittelgesetz)
AMRadV: Verordnung uber Radioaktive oder mit ionisierenden Strahlen behandelte Arzneimittel
ANSM: National Agency for the Safety of Medicines and Health Products
ARS: Regional Health Agency
AT: Austria
BASG: Austrian Federal Office for Safety in Health Care
BE: Belgium
BfArM: Federal Institute for Drugs and Medical Devices
BPP: Bonnes Practiques de Préparation
cGRPP: current Good Radiopharmaceutical Practice
CSP: Public Health Code
DK: Denmark
DMA: Danish Medicines Agency
EANM: European Association of Nuclear Medicine
EC: European Commission
EMA: European Medicine Agency
EPRPs: Extemporaneus Radiopharmaceutical preparations
EU: European Union
FI: Finland
FIMEA: Finnish Medicines Agency
GMP: Good Manufacturing Practices
GPP: Guide to Good Practices
IMP: Investigational Medicinal Product
IMPD: Investigational Medicinal Product Dossier
IT: Italy
MA: Marketing Authorization
MHRA: Medicines & Healthcare Products Regulatory Agency
MIA: Marketing Importation Authorization
NBP-MN: Norme di buona preparazione in medicina nucleare
NC: Not confirmed
PET: Positron Emission Tomography
PIC/S: Pharmaceutical Inspection Co-operation Scheme
PUI: pharmacie à l’usage intérieur
RD: Real Decree
RLD: Royal Legislative Decree
RPs: Radiopharmaceuticals
SPECT: Single Photon Emission Tomography
UK: United Kingdom
