# Supplementary material for: Radiopharmaceutical small-scale preparation in Europe: will we be able to harmonize the situation?
Source: EJNMMI Radiopharm Chem. 2024 Sep 5;9:64. doi: 10.1186/s41181-024-00281-z (PMC11377369; doi:10.1186/s41181-024-00281-z)
Supplement: Supplementary file 1 — Supplementary Material 1 [file 41181_2024_281_MOESM1_ESM.docx]

José Antonio Guerra Guirao

Universidad Complutense de Madrid

Pz Ramón Y cajal s/n. 28040. Madrid. Spain

Tel. +34-ext.913941769; e-mail: [jaguerra@ucm.es](mailto:jaguerra@ucm.es)

Editor

European Journal of Nuclear Medicine and Molecular Imaging

June 5th, 2024

Dear Editor Philip H. Elsinga,

Thank you for editing our manuscript “*Radiopharmaceutical small-scale preparation in Europe. Will we be able to harmonize the situation?*”, by Estrella Moya; Cerrato, Celia Cerrato, Luis Miguel Bedoya and José Antonio Guerra, for consideration to be published as a review in *European Journal of Nuclear Medicine and Molecular Imaging.*

We really appreciate these new comments “ *it* *seems that you have correctly addressed the suggested revision in your cover letter, but for some reasons you did not change the text in the manuscript accordingly”.*

We believe there was a problem with the upload, as it appears that only part of the comments have been updated. We will re-upload the files (clean and track) with all the changes requested last time, as reflected in this cover letter.

As requested, we provide below point-by-point answers to the referee’s remarks. We thank the referee for his/her criticism, and hope that the new revised version of the manuscript will be suitable for publication in European Journal of Nuclear Medicine and Molecular Imaging.

Sincerely yours

José Antonio Guerra

Estrella Moya

On behalf of the authors

**Response to referees:**

**Reviewer 1**:

Suggested to correct "they can be considered as a magistral formula" with "they can be considered as a magistral and officinal formula" at page 14, line 34 (in the new version I assume is at page 13, line 30); it seems to me that the above requested correction has not been addressed and I would suggest to do that, as officinal formula approach is usually of more concern compared with magistral formula and should be included.

**Author’s response**: We appreciate the reviewer's suggestion. In the first revision, we had made the change in the Italy section, but we did not do it on page 13, line 30. We have changed the text as follows: *“In some Member States (France, Italy, Belgium) the unlicensed small-scale radiopharmaceutical preparations follow Article 3 of Directive 2001/83/EC and they can be considered as magistral formulas and officinal formulas”*

- this comment is mine: as for the table 3, column "compliance with GMP", I see two problems: i) in my opinion it is not correct to talk about "GMPs"; as you are considering the EU regulatory landscape, in the EU there are not GMPs (plural) but only one GMP, so I would clearly state that GMP are to be intended as official EU GMP; As GMPs (plural) is frequently recurring throughout the text, I would correct all the occurrences from "GMPs" to "GMP" ; ii) in the same column of table 3, you wrote "GMPs" for Italy, Spain and UK, but if you mean they have to follow GMP then it's better to write "Yes" (as you did for denmark and germany) or "no"; By the way, in case of Italy I am sure that the correct answer is "no", as NBP-MN are applicable and not GMP

**Author’s response**: We acknowledge the reviewer‘s remark and we are in full agreement to use the official term in the EU. We have changed all the occurrences from "GMPs" to "GMP.

In addition, in Table 3, we have modified the table according to the reviewer's suggestions. In the case of Italy, we had not realized that there was a mistake, the correct answer is no, as the reviewer has noted.

**- Reviewer 2**

Suggested some corrections to the description of ETH courses / EANM postgraduated courses; the description you included in the revised version of the manuscript is correct in the first part (until page, 13, beginning of line 9), related to ETH courses, but it is wrong in the second part, related to EANM post graduated course. Indeed, as reviewer 2 stated in his comment, EANM is not organizing any course, but simply recognizing the CAS certificate released by ETH and then awarding students with EANM Certification provided that also other requirements are met (see below). I suggest to look at the following web page "<https://esmit.eanm.org/radiopharmacy-certification/>", where you can find proper information, that may be summarized as follows: i) the radiopharmacy courses are organized only by ETH, which at the end release a CAS; ii) EANM may then release the post-graduate certification provided that candidates have successfully completed the above ETH courses and obtained the related CAS, but they also have to demonstrate to have completed a two-year period of experience in a radiopharmacy department, during which they have completed the practical components of the ETH course syllabus, and completed a nationally recognised course on radiation safety. I hope it's now more clear and that you may correct the manuscript properly.

**Author’s response**: We really appreciate the reviewer's clarification. We have therefore reworded the paragraph in page 15 as follows:

*“The ETH courses are recognized by the EANM, which may issue the postgraduate certificate to candidates who have successfully completed them and obtained the corresponding CAS, but who must also demonstrate that they have completed a two-year period of experience in a radiopharmacy department, during which they have completed the practical components of the ETH course syllabus, and have completed a nationally recognized course in radiation safety”.*

- about one of the comments of reviewer 2 about specialization in France, you wrote (page 7) "In France, radiopharmacy is considered to be a pharmaceutical specialization, which can be obtained either through postgraduate studies in radiopharmacy: Diploma of Specializsed Studies in Radiopharmacy and Radiobiology (Diplôme d'etudes Spécialisées)." is not clear whether there are two different courses (postgraduate studies in radiopharmacy" and "diploma of specialized studies....", or if it is only one. If the correct interpretation is the second, please remove "either" and rewrite in a more clear way.

**Author’s response**: We acknowledge the reviewer‘s remark , the correct interpretation is “it is only one”. . We think it would be better to rewrite the paragraph more clearly, as suggested by the reviewer. The text is reworded as follows:

*“The person in charge of preparing the RP should be a radiopharmacist. In France, radiopharmacy is a specialty of pharmacy internship leading to a Diploma of Complementary Specialized Studies (DESC) of Radiopharmacy and Radiobiology, which comes in addition to the diploma of pharmacist former intern of hospital”.*

We also realized that there was an error in the following paragraph on page 15:

*“On the other hand, in the case of Spain and France, there is an official certificate to be considered a radiopharmacist specialist. In both countries, a specialization in pharmacy or chemistry is obtained through* ***three years of practical*** *experience in hospital and radiopharmacy industry. In these countries, other previously mentioned certificates are not legally recognized*.”

-Rationale:

The radiopharmacy specialization is Spain last three years, however in France is only two years of the total 5 years of pharmacy internship. We have rewritten the text as follows:

*“On the other hand, in the case of Spain and France, there is an official certificate to be considered a specialist in radiopharmacy. In both countries, the specialization in radiopharmacy is obtained through a period of practical experience in radiopharmacy in hospitals and in the radiopharmaceutical industry (3 years in Spain and 2 years in France). Other certificates mentioned above are not legally recognized in these countries”.*

- another comment from reviewer 2, requesting to correct "verordnung yesber" to verordnung uber": it has been correctect at page 9, but not in the abbreviation list at page 20

**Author’s response**: We acknowledge the reviewer‘s remark and we have corrected the abbreviation list.
